# An affordable and easy-to-manage hydroponic system to cultivate hundreds of rice plants in a small space

**DOI:** 10.17912/micropub.biology.002005

**Published:** 2026-07-26

**Authors:** Yoshihiro Hase, Yong-Gen Yin, Nobuo Suzui, Naoki Kawachi, Pronabananda Das, A.N.K. Mamun

**Affiliations:** 1 Takasaki Institute for Advanced Quantum Science, National Institutes for Quantum Science and Technology, Chiba, 12, Japan; 2 Institute of Food and Radiation Biology, Bangladesh Atomic Energy Commission, Savar, Dhaka, Bangladesh

## Abstract

We developed a hydroponic system using readily available materials. This system enables uniform and quick cultivation of hundreds of rice plants with simple management. Uniform cultivation of more than 1,000 rice plants in a 1 m × 2 m space was achieved mainly by reducing the plant height with growth retardant and optimizing the arrangement of plants. Heading time under this system may vary depending on photoperiod sensitivity or other genotype-specific responses. However, under appropriate conditions, this method will be useful not only for generation advancement, but also for screening mutants under specified cultivation conditions.

**Figure 1. Hydroponic system to cultivate hundreds of rice plants in a small space:** (A) Hydroponic culture system constructed using commercially available materials. Red arrows indicate flow of hydroponic solution. (B) Rigid polyurethane foam with 180 holes to hold 15-ml tubes containing rice plants. (C) Two-week-old seedlings grown using a 96-well PCR plate with the bottom cut off. (D) Plants 30 days after sowing (2-week-old seedlings as starting materials, followed by 16 days of cultivation using hydroponic system). (E) Changes in pH of hydroponic solution. Green triangles indicate day when nutrients were added. Red asterisk indicates heading date of rice ( *Oryza sativa* L. cv. Nipponbare). (F) Representative Nipponbare plants at maturity stage. Bar = 5 cm. Values indicate mean ± standard deviation and range. (G) Nipponbare and BRRIdhan47 plants at 90 days after sowing. (H) Six trays arranged in three tiers to cultivate more than 1,000 rice plants in 1 m × 2 m space.

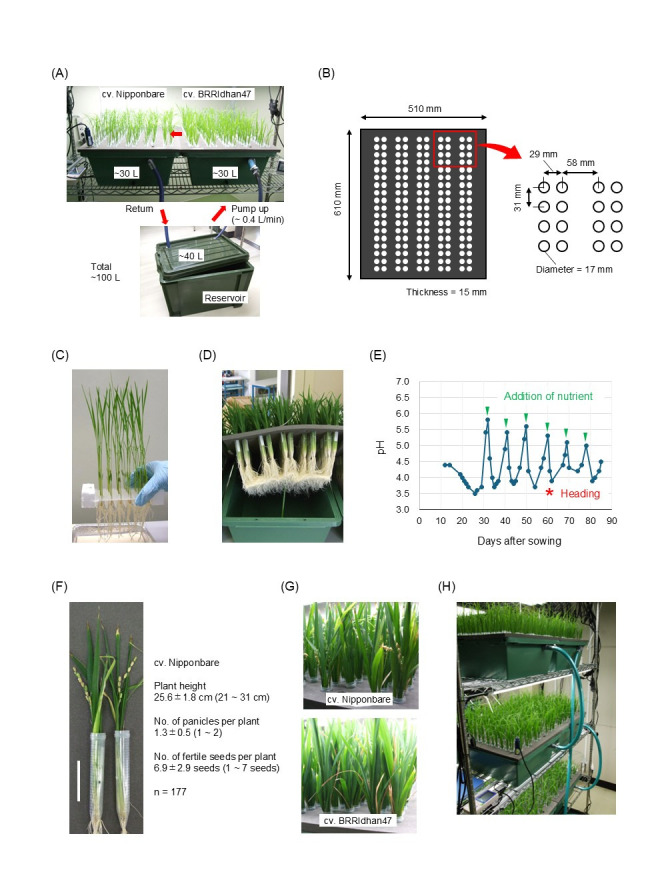



## Description

To obtain useful mutant lines of rice, hundreds to thousands of mutagenized plants must be screened. Rapid generation advancement is also essential for rice research and breeding. Hydroponic systems have been developed using commonly available plant incubators to rapidly cultivate dozens of rice plants (Kuroda and Ikenaga, 2015; Tanaka et al., 2016). However, practical application of these systems on a large scale is difficult because several incubators are required and maintaining uniform hydroponic conditions across them is challenging. For generation advancement and for mutant screening, it would be useful if hundreds of rice plants could be uniformly cultivated in a small space with a hydroponic system under specified cultivation conditions, even if only a small number of seeds can be obtained from individual plants. To this end, we have developed an affordable and easy-to-manage hydroponic system constructed from readily available materials.

The cultivation system comprises two plastic trays and a reservoir tank connected to each other ( Figure 1A ). Approximately 100 L hydroponic solution in total was circulated using an aquarium pump with a flow rate of approximately 0.4 L/min. The hydroponic solution was prepared using tap water and 1/80 strength Murashige and Skoog basal salts as described in the Methods (Table 1). The initial pH value was adjusted to around 4.5 with hydrochloric acid. The total amount of hydroponic solution gradually decreased as the plants grew, while the water level in the plastic trays was kept constant. Deionized water was added to the reservoir tank once a week. A rigid polyurethane foam with 180 holes was placed on the top of each plastic tray to hold 15-ml plastic tubes with the bottoms cut off ( Figure 1B ). Uniform cultivation is difficult when many plants are cultivated at a high density, because plants located in the center of a population receive insufficient light to grow compared with those in the border because of the ‘border effect’ (Sun et al., 2023). To overcome this problem, we added Uniconazole-P, a gibberellin biosynthesis inhibitor (Saito et al., 2006), to reduce the plant height.

Two rice cultivars were used: ‘Nipponbare’, a representative japonica cultivar; and ‘BRRIdhan47 ’, a salt-tolerant indica cultivar developed in Bangladesh (Fahim et al., 2010). Dry seeds were placed in the wells of a 96-well PCR plate, the bottoms of which were removed. The seeds floated on tap water for 1 week to allow them to germinate. The germinated seeds were grown for a further week with the hydroponic solution ( Figure 1C ). The 2-week-old seedlings were then inserted into 15-mL tubes immobilized in the polyurethane foam. It was not necessary to pull out the roots from the bottom of the 15-ml tubes, because new roots soon developed from the basal nodes of the seedlings ( Figure 1D ). In total, 177 seedlings were grown for each cultivar ( Figure 1A ). The cultivation system was placed in a room controlled at around 27 °C. The plants were grown under short-day conditions (10-h light /14-h dark), with light supplied by white light emitting diode (LED) lights. Figure 1E shows the change in the pH of the hydroponic solution throughout the growth period. The hydroponic solution contained both ammonium ions and nitrate ions. Therefore, the pH value decreased as the rice plants preferentially absorbed ammonium ions, and then increased after the plants started to absorb nitrate ions. The initial amount of nutrients, except growth retardant, was added when the pH value increased beyond 5.0 ( Figure 1E ). In this manner, the pH value was kept within the approximate range of 4.0 to 5.5, the range preferable for rice plant growth, without any manual adjustment using acid or alkali.

The Nipponbare plants started heading 60 days after sowing and the seeds matured before 90 days after sowing ( Figure 1E and F). This generation time is comparable to those reported in prior studies (Kuroda and Ikenaga, 2015; Tanaka et al., 2016). The plant height was 25.6 ± 1.8 cm (mean ± standard deviation, *n* = 177). Although the fertility was not very high ( Figure 1F ), almost all plants (176 out of 177) produced fertile seeds. The average number of fertile seeds per plant was 6.9 ± 2.9 (mean ± standard deviation, *n* = 176).

By contrast, none of the BRRIdhan47 plants had reached the heading stage at 90 days after sowing, the time when all the fertilized Nipponbare seeds had matured ( Figure 1G ). Several BRRIdhan47 plants started heading at 123 days after sowing, but most of the BRRIdhan47 plants still had no signs of heading. This was likely because of the specific growth conditions of the hydroponic system, because both cultivars started heading at a similar time when they were grown outdoors in pots with soil under natural conditions from spring through autumn in Gunma prefecture, Japan. A previous study showed that among 48 rice accessions, the days to heading (DTH) under hydroponic culture with high-temperature, short-day conditions tended to be significantly fewer than the corresponding DTH under field conditions (Tanaka et al., 2016). However, the difference was sometimes small, and one of those accessions showed longer DTH under hydroponic conditions than under field conditions. Thus, this hydroponic system is likely to be suitable for cultivation of many rice strains, except some specific strains including BRRIdhan47.

The hydroponic system described here is easily expandable. More than 1,000 rice plants can be grown and harvested in a 1 m × 2 m space by arranging six trays in three tiers ( Figure 1H ). In this hydroponic system, the plant height is controlled by applying a growth retardant. This is indispensable to achieve uniform cultivation. The proper arrangement of plants, i.e., two rows of holes arranged with sufficient space between holes and rows ( Figure 1B ), is also essential so that the plants receive uniform light. Light intensity and the nitrogen concentration are also important factors because excess light or nitrogen stimulate tillering, resulting in reduced quality of mature seeds. If the correct conditions are met, this hydroponic system enables uniform and quick cultivation of hundreds of rice plants with simple management.

## Methods

**Hydroponic culture system**

Plastic trays (capacity 36 liter, W610 × D468 × H185 mm) and plastic containers (43 liter, W379 × D545 × H322 mm) were connected using a water hose and PVC pipes, which circulated the hydroponic solution. The plastic containers were placed on wire shelves ( Figure 1A ). The culture solution was circulated using an aquarium pump (18 W). Rigid polyurethane foam (15-mm thick) was cut into a 510 × 610 mm rectangle, and 180 holes with a diameter of 17 mm were made using a cork borer ( Figure 1B ). The bottoms of 15-ml conical tubes were cut off, and the bottomless tubes were inserted into the holes of the polyurethane foam. Ten straight-tube white LED lamps (22 W) were attached to the wire shelf, and their position was adjusted so that the light intensity was as uniform as possible. Light was controlled with a 24-h timer, and the light intensity was 150–200 μmol/m ^2^ /s at 10 cm above the polyurethane foam.

**Culture solution**

Hydroponic culture solution was prepared using tap water and 1/80 strength Murashige and Skoog basal salts supplemented with microelements and ferric ions (Table 1). The initial pH value was adjusted to around 4.5 with hydrochloric acid. Sumiseven P (0.025% Uniconazole P, Sumitomo Chemical Co., Ltd.) was added at a dilution rate of 15,000 times (final concentration of Uniconazole P = 1.67 × 10 ^-6^ %).

**Table 1.** Composition of nutrient solution.

**Table:** 

**Macro elements**	**Concentration (mg/L)**
NH _4_ NO _3_	20.6
KNO _3_	23.8
CaCl _2_ −2H2O	5.5
MgSO _4_ −7H _2_ O	4.6
KH _2_ PO _4_	2.1
**Microelements (× 4,000 stock)**	**Concentration (mg/L)**
MnSO _4_ −5H _2_ O	1.1
H _3_ BO _3_	0.93
ZnSO _4_ −7H _2_ O	0.043
CuSO _4_ −5H _2_ O	0.040
Na _2_ MoO _4_ −2H _2_ O	0.024
**Fe (× 4,000 stock)**	**Concentration (g/L)**
FeSO _4_ −7H _2_ O	3.72
Na _2_ -EDTA	2.78
**Growth retardant**	**Concentration (%)**
Uniconazole P	1.67 × 10 ^-6^
